# *Staphylococcus caprae* as an Emerging Pathogen in Infective Endocarditis: A Case Series and Review of the Literature

**DOI:** 10.1155/crdi/9983702

**Published:** 2026-04-16

**Authors:** Joel Isai Alcala-Gonzalez, Reynaldo Lara-Medrano, Karla Paola Cuellar-Calderon, Adrian Camacho-Ortiz

**Affiliations:** ^1^ Infectious Diseases Department, Hospital Universitario “Dr. José Eleuterio González”, Universidad Autónoma de Nuevo León, Monterrey, Mexico, uanl.mx

## Abstract

*Staphylococcus caprae*, a non‐*aureus staphylococcus* primarily associated with goats, is typically regarded as a commensal organism. While its role in veterinary contexts is well established, its emergence as a human pathogen in healthcare settings has gained increasing recognition. It has been implicated in infections such as bacteremia, otitis externa, osteoarticular infections, and prosthetic device–related complications. However, infective endocarditis caused by *S. caprae* remains extremely rare, with only six cases reported in the literature since 1995. This report presents a case of native valve infective endocarditis due to methicillin‐resistant *S. caprae* in a previously healthy 40‐year‐old male with no history of animal contact. The patient presented with fever, dyspnea, and signs of heart failure. Echocardiographic evaluation revealed vegetations on both the aortic and mitral valves, and blood cultures confirmed the presence of *S. caprae*. Initial empirical therapy with ceftriaxone and vancomycin was started and later adjusted to linezolid based on antimicrobial susceptibility testing. Despite appropriate antimicrobial treatment and intensive supportive care, the patient’s condition progressively deteriorated. He experienced neurological decline, hemodynamic instability, and ultimately died following cardiac arrest before surgical intervention could be performed. This case highlights the diagnostic challenges in identifying *S. caprae*, as conventional phenotypic methods often misclassify the organism. Advanced techniques such as MALDI‐TOF MS provide more accurate identification. Antimicrobial susceptibility testing is essential, as treatment options may vary. Fosfomycin resistance may serve as a presumptive marker for *S. caprae*, though cross‐resistance with other species limits its specificity. Given the rarity of *S. caprae* endocarditis, clinicians should maintain awareness of its potential occurrence, even in patients without animal exposure but with healthcare‐associated risks. Early microbiological diagnosis, targeted antibiotic therapy, and prompt surgical evaluation are crucial for managing this uncommon and clinically significant infection.

## 1. Introduction

Staphylococci are significant components of the normal microbiota among humans and animals [1]. Most skin commensals, including non‐*aureus staphylococcus* (NAS), rarely pose a threat to healthy individuals. Instead, they contribute to defense by preventing the invasion of more pathogenic or harmful organisms [2].

*Staphylococcus caprae* is one of the animal‐associated NAS that usually colonizes goats’ skin and mammary glands and sometimes causes goat mastitis. However, *S. caprae* infections have recently been also recognized as hospital‐acquired infections since most *S. caprae* infections were related to medical care and contracted during hospital stay [3]. Well‐documented *S. caprae* infections include bacteremia, acute otitis externa, bone and joint infections, and prosthetic infections [4, 5].

*S. caprae* endocarditis is a rare event with only a few cases registered in the medical literature; thus, we present a case of infective endocarditis involving aortic and mitral native valves in a patient without animal contact and perform a literature review.

### 1.1. Informed Consent

Written informed consent was obtained from the patient’s wife for the publication of this case report and any accompanying images. As the patient is deceased, the wife has provided consent as the legal next of kin.

### 1.2. Patient Information

In September 2024, a 40‐year‐old male, with a 2‐month history of fever and dyspnea, was admitted. He was from the south of Mexico but moved to the northwest 10 years ago. He had no relevant past medical history. Physical examination revealed bilateral lung base crackles, aortic diastolic murmur III/IV, and edema in lower limbs. An EKG revealed left ventricle hypertropia and left bundle branch block. Two sets of aerobic blood cultures were positive for methicillin‐resistant *S. caprae* (phenotypic identification was carried out using the Vitek 2 system with a GP ID card [bioMérieux, Marcy l’Etoile, France]). Transthoracic echocardiography plus transesophageal echocardiogram (TEE) confirmed a bicuspid aortic valve and vegetation in the same valve of 11 × 14 mm, causing severe insufficiency and mild stenosis. Vegetation was also reported on the anterior mitral valve (Figure 1).

FIGURE 1Transthoracic echocardiogram, parasternal long axis. (a) Vegetation present on the aortic valve. (b) Aortic insufficiency evidenced by Doppler color. LA: left auricle. LV: left ventricle. RV: right ventricle. Ao: aorta. ^∗^Vegetation in the aortic valve.

**Figure:**
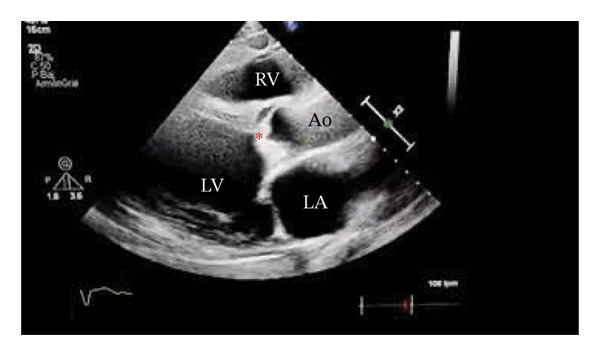
(a)

**Figure:**
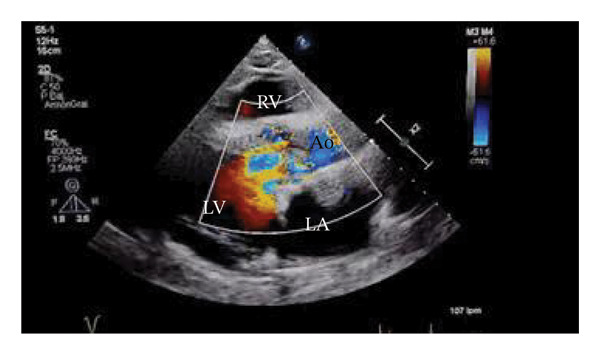
(b)

The patient was admitted to the ICU and received supportive treatment for acute heart failure, with urgent surgery scheduled.

Empirical antimicrobial therapy was initiated with ceftriaxone (1 g every 12 h, IV) and vancomycin (30 mg/kg/day, ID divided in 2 doses). After *S. caprae* was identified and susceptibility testing was performed (Table 1), antimicrobial therapy was adjusted to linezolid (600 mg every 12 h, IV) as monotherapy.

**TABLE 1:** Results of antibiotic sensitivity data.

Isolation from blood culture: *Staphylococcus caprae*		
Antibiotic	mcg/mL	Interpretation
Clindamycin	> 4	Resistant
Daptomycin	≤ 0.5	Susceptible
Erythromycin	≤ 0.5	Susceptible
Linezolid	2	Susceptible
Oxacillin	0.5	Resistant
Penicillin	> 8	Resistant
Rifampin	≤ 1	Susceptible
Trimethoprim/sulfamethoxazole	≤ 0.5/9.5	Susceptible
Tetracycline	≤ 4	Susceptible
Vancomycin	1	Susceptible

Two days later, the patient experienced neurological deterioration and required intubation. Despite ongoing antibiotic therapy, the aortic vegetation continued to worsen, severely compromising his hemodynamic stability. He subsequently suffered a cardiac arrest due to ventricular tachycardia. After four cycles of resuscitation and spontaneous circulation was restored, a subsequent cardiac arrest ultimately ended his life. Unfortunately, it was not possible to obtain control blood cultures to confirm microbiologic clearance.

## 2. Discussion

Infective endocarditis caused by of *S. caprae* is exceptionally rare; since 1995, only six cases have been reported in the medical literature [6–10].

Conventional phenotypic identification commonly underestimates the prevalence of *S. caprae*. Some automated phenotypic identification systems misclassify many clinical strains, making them more challenging to diagnose; Allignet et al. reported the 50% identification of *S. caprae* strains in bone and join infections with ID32‐staph system (BioMérieux, Marcy l′Etoile, France) [5]. Another identification systems such as MicroScan (Dade Behring, West Sacramento, CA, USA), Vitek 2 (BioMérieux), and Crystal GP (Becton Dickinson, Sparks, MD, USA) also misclassify these strains [11].

PCR methods are specific for detecting microorganisms that cause various infectious diseases and are good at discriminating between different related species. Currently, PCRs used to detect 16S rRNA genes are not sufficient to differentiate species such as *S. caprae*, *S. capitis*, and *S. saccharolyticus*, as they share a high degree of sequence similarity (> 97%) with *S. epidermidis*. Other protein targets have been studied (such as the Gram‐positive signal peptide protein), as well as techniques using specific sequences and primers integrated into multiplex PCR to differentiate *S. caprae* from the other members of the *S. epidermidis* group (*S. capitis* and *S. saccharolyticus*). However, these techniques are not yet readily available in every laboratory [12, 13].

Susceptibility to antimicrobial agents may aid, and resistance to fosfomycin may be a presumptive test for identifying *S. capra*e; however, several other staphylococcal species have intrinsic resistance to this antibiotic (*S. saprophyticus*, *S. hominis*, *S. capitis*, *and S. auricularis*) [5].

Matrix‐assisted laser desorption ionization time‐of‐flight mass spectrometry (MALDI‐TOF MS) for bacterial identification in the clinical microbiology laboratory has been shown to accurately identify clinical *S. caprae* strains [14].

This organism has been reported to colonize the nose, nails, and skin, principally in those persons that are or have been in contact with animals. Although there are multiple reports of osteoarticular infections caused by this organism in the literature, information regarding its association with infective endocarditis is limited [5, 9].

The first reported case of infective endocarditis occurred in 1995, involving the mitral valve. It was managed with early surgical removal of significant vegetation while preserving the native valve, followed by 2 weeks of intravenous vancomycin, resulting in a favorable recovery. Subsequently, we identified five additional cases, half of which involved patients with underlying cardiopathy but without prosthetic valve material. Antimicrobial regimens varied though beta‐lactams were primarily used, followed by vancomycin (Table 2).

**TABLE 2:** Summary characteristics of *Staphylococcus caprae* infective endocarditis described in the literature.

Case	Case 1	Case 2	Case 3	Case 4	Case 5	Case 6	Case 7 (presented)
Year of publication	1995 [6]	2016 [7]	2020 [8]	2023 [9]	2023 [9]	2024 [10]	2024
Age (y)	46	76	45	73	50	53	40
Sex	Male	Male	Male	Male	Female	Male	Male
Animal contact	Not reported	No	Goats	Goats	Pig slaughterhouse	Not reported	No
Underlying cardiomyopathy	No	Mild mitral regurgitation	No	Aortic prothesis	Tetralogy of Fallot and pulmonary prothesis	No	Bicuspid aortic valve
Type of IE and valve involved	Native, mitral	Native, mitral	Native, aortic	Prosthetic, aortic; native, mitral	Prosthetic, pulmonary	Native, mitral	Native, aortic, and mitral
Embolism	None	Brain	Kidney, spleen, myocardial infarction	None	Lung	Brain	Not documented
Diagnostic specimen	Blood culture, valve culture	Blood culture	Valve culture	Blood culture, autopsy	Blood culture	Blood culture	Blood culture
Microbiologic characteristic							
Penicillin MIC (μg/mL)	Resistant MIC not reported	Resistant MIC not reported	Not reported	Susceptible. MIC ≤ 0.03	Resistant. MIC ≥ 0.5	Susceptible MIC not reported	Resistant. MIC > 0.8
Cloxacillin MIC (μg/mL)	Susceptible MIC not reported	Susceptible MIC not reported	Resistant MIC not reported	Susceptible. MIC = 0.5	Susceptible. MIC ≤ 0.25	Susceptible MIC not reported	Resistant. MIC ≥ 0.5
Vancomycin MIC (μg/mL)	Susceptible MIC not reported	Susceptible MIC not reported	Susceptible MIC not reported	Susceptible. MIC = 1	Susceptible. MIC ≤ 0.5	Susceptible MIC not reported	Susceptible. MIC = 1
Treatment	Vancomycin (2 weeks)	Flucloxacillin (6 weeks)	Vancomycin (4 weeks)	Cloxacillin, rifampicin (6 weeks), gentamicin (2 weeks)	Cloxacillin, rifampicin (6 weeks) followed by levofloxacin, rifampicin	Vancomycin and ciprofloxacin (2 weeks)	Ceftriaxone and vancomycin, once ID was obtained, linezolid.
Surgery	Vegetectomy	No	Yes	Yes	No	No	No
Outcome	Survived (at 3 y)	Survived (at 3 m)	Survived	Died	Survived (at 9 m)	Died	Died

Despite supportive care and antimicrobial therapy, it was clear that the patient required surgical intervention. The size of the vegetation and the extent of hemodynamic compromise made him a candidate for valve replacement. Antimicrobial treatment should be based on susceptibility patterns as no standardized guidelines exist. The use of different antibiotics has been documented, but recommendations are limited and depend on local experience.

## 3. Conclusion

*S. caprae* infections are infrequent, with infective endocarditis representing an exceptionally rare manifestation. Clinical suspicion should be maintained not only in individuals with a history of animal contact but also in those with potential exposure to healthcare‐associated environments. Early microbiological identification, initiation of targeted antimicrobial therapy, and prompt involvement of cardiac surgical teams are critical, given the high morbidity and mortality associated with this pathogen.

## Funding

This research did not receive any specific grant from funding agencies in the public, commercial, or not‐for‐profit sectors.

## Conflicts of Interest

The authors declare no conflicts of interest.

## Data Availability

Data sharing is not applicable to this article as no datasets were generated or analyzed during the current study.

## References

[1] Evans C. A. , Smith W. M. , Johnston E. A. , and Giblett E. R. , Bacterial Flora of the Normal Human Skin, Journal of Investigative Dermatology. (1950) 15, no. 4, 305–324, 10.1038/jid.1950.105, 2-s2.0-0142235149. 14779047

[2] Grice E. A. and Segre J. A. , The Skin Microbiome, Nature Reviews Microbiology. (2011) 9, no. 4, 244–253, 10.1038/nrmicro2537, 2-s2.0-79952750288. 21407241 PMC3535073

[3] Watanabe S. , Aiba Y. , Tan X.-E. et al., Complete Genome Sequencing of Three Human Clinical Isolates of Staphylococcus caprae Reveals Virulence Factors Similar to Those of *S. epidermidis* and *S. capitis* , BMC Genomics. (2018) 19, no. 1, 10.1186/s12864-018-5185-9, 2-s2.0-85056405009. PMC6225691 30409159

[4] Seng P. , Barbe M. , Pinelli P. O. et al., *Staphylococcus caprae* Bone and Joint Infections: A Re-Emerging Infection?, Clinical Microbiology and Infection. (2014) 20, no. 12, O1052–O1058, 10.1111/1469-0691.12743, 2-s2.0-84920432579. 24975594

[5] Allignet J. , Galdbart J.-O. , Morvan A. et al., Tracking Adhesion Factors in *Staphylococcus caprae* Strains Responsible for Human Bone Infections Following Implantation of Orthopaedic Material, Microbiology. (1999) 145, no. 8, 2033–2042, 10.1099/13500872-145-8-2033, 2-s2.0-0032869759. 10463169

[6] Vandenesch F. , Eykyn S. J. , Bes M. , Meugnier H. , Fleurette J. , and Etienne J. , Identification and Ribotypes of *Staphylococcus caprae* Isolates Isolated as Human Pathogens and From Goat Milk, Journal of Clinical Microbiology. (1995) 33, no. 4, 888–892, 10.1128/jcm.33.4.888-892.1995. 7790455 PMC228061

[7] Kwok T. C. , Poyner J. , Olson E. , Henriksen P. , and Koch O. , *Staphylococcus caprae* Native Mitral Valve Infective Endocarditis, JMM Case Reports. (2016) 3, no. 5, 10.1099/jmmcr.0.005065. PMC5343145 28348787

[8] Hammami R. , Ben Ali Z. A. , Charfeddine S. , Abid L. , and Kammoun S. , Endocardite Infectieuse À *Staphylococcus Caprae* Compliquée de Syndrome Coronarien Aigu, Medecine et Maladies Infectieuses. (2020) 50, no. 6, 531–533, 10.1016/j.medmal.2020.04.008. 32315703

[9] Díez De Los Ríos J. , Hernández-Meneses M. , Navarro M. , Montserrat S. , Perissinotti A. , and Miró J. M. , Staphylococcus Caprae: An Emerging Pathogen Related to Infective Endocarditis, Clinical Microbiology and Infection. (2023) 29, no. 9, 1214–1216, 10.1016/j.cmi.2023.06.006. 37321397

[10] Zhu Z. , Li Y. , and Yu X. , A Case Report of Infective Endocarditis Caused by Staphylococcus caprae, Case Reports. (2024) 52, no. 11, 1324–1326, 10.3760/cma.j.cn112148-20231213-00492. 39557534

[11] Kim M. , Heo S. R. , Choi S. H. et al., Comparison of the Microscan, VITEK 2, and Crystal GP with 16S rRNA Sequencing and MicroSeq 500 v2.0 Analysis for coagulase-negative Staphylococci, BMC Microbiology. (2008) 8, no. 1, 10.1186/1471-2180-8-233, 2-s2.0-60149104069. PMC2633347 19105808

[12] Kim E. , Yang S.-M. , Won J.-E. , Kim D.-Y. , Kim D.-S. , and Kim H.-Y. , Real-Time PCR Method for the Rapid Detection and Quantification of Pathogenic Staphylococcus Species Based on Novel Molecular Target Genes, Foods. (2021) 10, no. 11, 10.3390/foods10112839. PMC8618141 34829120

[13] Hirotaki S. , Sasaki T. , Kuwahara-Arai K. , and Hiramatsu K. , Rapid and Accurate Identification of Human-Associated Staphylococci by Use of Multiplex PCR, Journal of Clinical Microbiology. (2011) 49, no. 10, 3627–3631, 10.1128/jcm.00488-11, 2-s2.0-80053494520. 21832022 PMC3187289

[14] Matsuda N. , Matsuda M. , Notake S. et al., Evaluation of a Simple Protein Extraction Method for Species Identification of Clinically Relevant Staphylococci by Matrix-Assisted Laser Desorption Ionization-Time of Flight Mass Spectrometry, Journal of Clinical Microbiology. (2012) 50, no. 12, 3862–3866, 10.1128/JCM.01512-12, 2-s2.0-84869220282. 22993187 PMC3502947

